# *Caprin* Controls Follicle Stem Cell Fate in the *Drosophila* Ovary

**DOI:** 10.1371/journal.pone.0035365

**Published:** 2012-04-06

**Authors:** John Reich, Ophelia Papoulas

**Affiliations:** The Section of Molecular Cell and Developmental Biology and the Institute for Cellular and Molecular Biology, The University of Texas at Austin, Austin, Texas, United States of America; University of Otago, New Zealand

## Abstract

Adult stem cells must balance self-renewal and differentiation for tissue homeostasis. The *Drosophila* ovary has provided a wealth of information about the extrinsic niche signals and intrinsic molecular processes required to ensure appropriate germline stem cell renewal and differentiation. The factors controlling behavior of the more recently identified follicle stem cells of the ovary are less well-understood but equally important for fertility. Here we report that translational regulators play a critical role in controlling these cells. Specifically, the translational regulator *Caprin* (*Capr*) is required in the follicle stem cell lineage to ensure maintenance of this stem cell population and proper encapsulation of developing germ cells by follicle stem cell progeny. In addition, reduction of one copy of the gene *fmr1,* encoding the translational regulator Fragile X Mental Retardation Protein, exacerbates the *Capr* encapsulation phenotype, suggesting *Capr* and *fmr1* are regulating a common process. Caprin was previously characterized in vertebrates as Cytoplasmic Activation/Proliferation-Associated Protein. Significantly, we find that loss of *Caprin* alters the dynamics of the cell cycle, and we present evidence that misregulation of *CycB* contributes to the disruption in behavior of follicle stem cell progeny. Our findings support the idea that translational regulators may provide a conserved mechanism for oversight of developmentally critical cell cycles such as those in stem cell populations.

## Introduction

**Figure 1:**
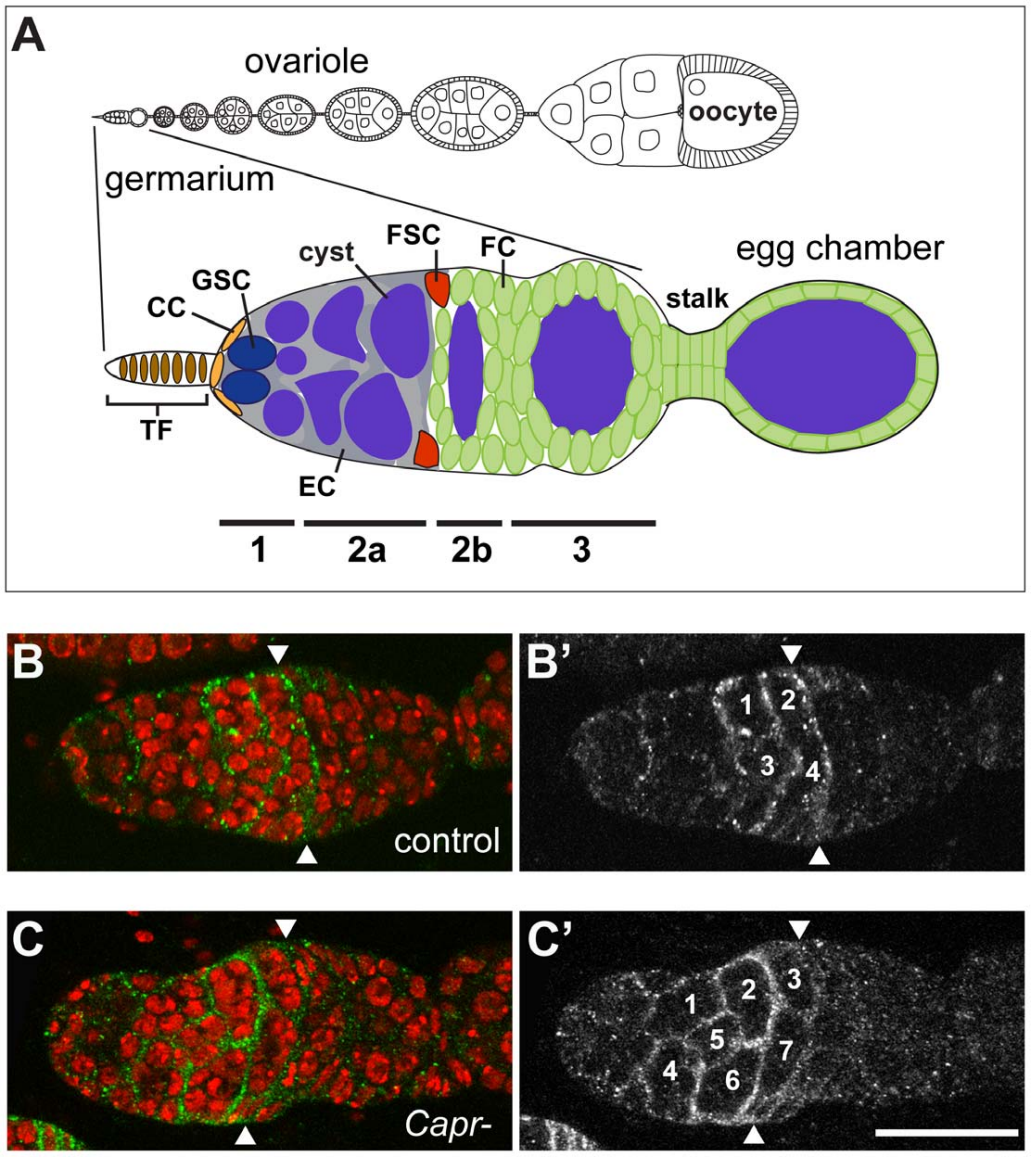
Loss of *Capr* disrupts germline cyst development. A) Schematic of the *Drosophila* germarium with bars below indicating the numbered germarium regions (1, 2a, 2b, 3) and their cell types: non-proliferating terminal filament (TF) and cap cells (CC), germline stem cells (GSC) which give rise to the developing 16-cell cysts (cyst), escort cells (EC) which facilitate movement of cysts through regions 1 and 2a, and the follicle stem cells (FSC) which give rise to the follicle cells (FC) and stalk. Anterior is to the left in all figures. B-C) Immunofluorescence analysis of control *+/Df(3L)Cat* (B, B’) or *Capr^2^/Df(3L)Cat* (C, C’) germaria stained with antibodies to Slit (green and B’, C’) and TO-PRO-3 iodide (DNA, red). Arrowheads indicate the position of FSCs and the unencapsulated cysts are numbered in B’ and C’. Scale bar is 30 microns.

Distinct stem cell populations within the ovary produce the different cell types that must act coordinately to create a functional egg. The *Drosophila* ovary has proved an extremely fruitful model system to study this process (reviewed in [1]). Two stem cell populations have been identified: the germline stem cells (GSCs), and the follicle stem cells (FSCs), which reside at the anterior of the ovariole in a structure called the germarium (Figure 1A). The GSCs give rise to the invariant 15 nurse cells and single oocyte comprising a cyst. Two FSCs produce all of the different types of somatic cells that surround the cysts and connect the developing egg chambers. During development, a cyst progresses through four morphologically and functionally distinct regions of the germarium: 1, 2a, 2b and 3 ([2] and Figure 1A). Region 1 houses the GSCs and escort cells [3], [4], [5]. Here, GSCs divide to produce another GSC (self renewal) and a cystoblast that undergoes four synchronous divisions to produce a 16-cell cyst [6]. As cysts develop, cellular processes from the escort cells surround them in regions 1 and 2a of the germarium and help move the cysts through this region [3], [7]. Two FSCs reside at the border of regions 2a and 2b and produce the follicle cells, stalk cells, and other somatic cells associated with a developing egg chamber [8], [9], [10]. Once a cyst is encapsulated it buds off from the germarium forming a stage 1 egg chamber. Production of a functional egg requires proper control of proliferation and differentiation of both stem cell populations and their progeny.

Stem cell activity is controlled by intrinsic and extrinsic factors, which operate in the context of specialized microenvironments, stem cell niches (reviewed in [1], [11]). Much is known about the molecular mechanisms regulating GSCs and their role in producing a functional egg (reviewed in [1]). For example, GSCs are found in a cellular niche at the anterior of the germarium. They are anchored to the cap cells via DE-cadherin, and loss of this adhesion leads to loss of stem cell properties [12]. In their niche, GSCs receive extrinsic signals, such as Dpp, from cap cells, that maintain their stem cell identity and prevent differentiation [13], [14]. Numerous intrinsic factors have also been identified that control GSC proliferation and differentiation and comprise a variety of molecular mechanisms. Prominent among them are proteins involved in translational regulation such as the eukaryotic initiation factor eIF4A and the translational regulators Pumilio, Nanos, and Vasa, [15], [16], [17], [18], [19] and components of the microRNA pathway [20], [21], [22], [23], [24]. In addition, GSC self-renewal and differentiation rely on chromatin modifiers which influence transcriptional regulation [25], [26]. Both intrinsic and extrinsic factors ensure that GSCs remain in an undifferentiated state while in their niche, yet continue to produce daughter cells that form the invariant 16-germ cells of each cyst.

Significantly less is known about the regulation of the FSCs. While FSCs also require cell adhesion proteins to maintain their stem cell identity, in this case DE-cadherin and integrins [12], [27], the cellular nature of the FSC niche is poorly understood. Recent work has suggested that each FSC may maintain contact with a single escort cell [7] however, the full complement of cells that comprise the FSC niche remains uncertain (reviewed in [1]). Like GSCs, FSCs also receive extrinsic signals controlling their proliferation and differentiation. These include long-range Hh and Wg signals, which emanate from the cap cells, and short-range signals from escort cells [28], [29], [30], [31], [32], [33]. Proteins modulating chromatin structure also appear to affect FSC self-renewal [25], [26], [34], [35]. To date, however, Dicer-1 is the only translational regulator identified as necessary for FSC maintenance or function [22]. Here, we report that the translational regulators Caprin (CAPR) and the *Drosophila* ortholog of Fragile X Mental Retardation Protein (FMRP) function together in regulating the FSC lineage. In addition, we find that FSC-lineage cells have an altered cell cycle in *Capr* mutants, further implicating *Capr* in developmental regulation of the cell cycle.

## Results

### Loss of *Capr* Produces Defects in Germline Cyst Packaging and Stalk Morphology

During our previous study [36] it was noted that *Capr-* females that were heterozygous for the *fmr1* gene (*Df(3L)Cat fmr1^3^/Capr^2^*) had reduced fecundity that decreased further with age (Figure S1). *fmr1* had been previously reported to have an extrinsic role in ovarian germline stem cell (GSC) maintenance [24], [37]. However, no germline phenotype was observed in heterozygous *fmr1* mutant ovaries, suggesting that the reduction of fecundity in *Df(3L)Cat fmr1^3^/Capr^2^* females was caused by loss of *Capr* or a combined requirement for *Capr* and *fmr1* in maintaining ovary function. To explore this possibility, ovaries from *Capr* null females were dissected and examined for morphological defects that could explain the contribution of *Capr* to the reduced fecundity. Initial observations indicated that *Capr^2^/Df(3L)Cat* mutant (hereafter referred to as *Capr-*) germaria were often swollen-looking and appeared to contain too many cells in region 2a. This phenotype could arise through hyperproliferation of cells within each cyst, or a local overabundance of morphologically normal cysts. Staining for the extracellular matrix protein Slit, which identifies escort and follicle cells in regions 2a and 2b of the germarium [10], revealed that compared to controls (Figure 1B, B’), there are an inappropriately high number of morphologically normal 16-cell cysts in 86% of *Capr-* germaria (Figure 1C, C’, and Table 1). Cyst production is controlled by proliferation and differentiation of GSC-lineage cells, while the follicle stem cell lineage is responsible for encapsulating cysts and mediating their exit from the germarium. The accumulation of cysts in region 2a of *Capr-* germaria could be due, therefore, to defects in either lineage.

**Table 1:** Loss of *Capr* increases the number of unencapsulated germline cysts.

Genotype	≤5 cysts	>5 cysts	n
*Df/+*	71.9%	28.1%	82
*Capr^2^/Df*	14.4%	85.6%	104
*ptc^S2^/+*	91.9%	8.1%	62
*ptc^S2^/+; Capr^2^/Df*	52.0%	48.0%	120
*wg^1–12^/+*	91.2%	8.8%	57
*wg^1–12^/+; Capr^2^/Df*	0.0%	100.0%	111
*CycB^2^/+; Capr^2^/Df*	49.5%	50.5%	95
*Act5C-GAL4; UAS-CycB*	21.6%	78.4%	88
*fmr1^3^/Df(3R)Exel6265*	81.4%	18.6%	59
*Df, fmr1^3^/Capr^2^*	0.0%	100.0%	91

The percent of total germaria scored (n) containing the normal number of Slit-stained 16-cell cysts (≤5 cysts), or supernumerary 16-cell cysts (>5 cysts), is shown for each genotype. *Df* refers to the *Capr* deficiency, *Df(3L)Cat*.

**Figure 2:**
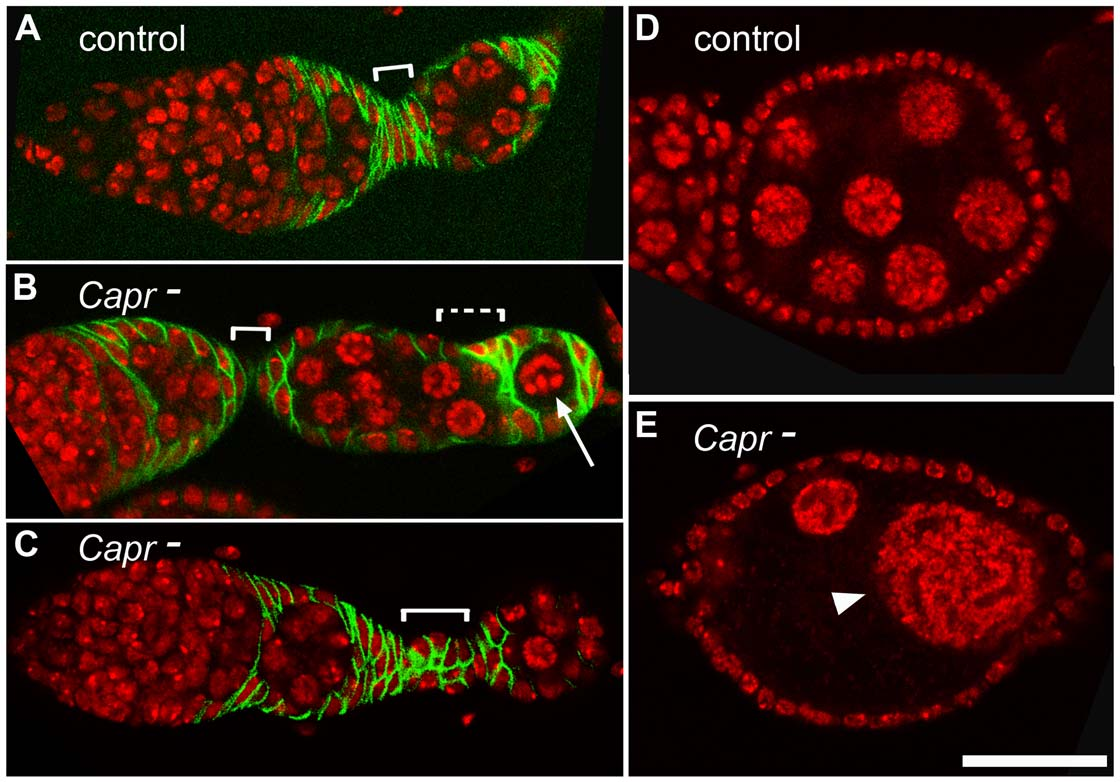
Characterization of egg chamber and stalk defects in *Capr*- ovaries. A-C) Germarium, budding egg chamber, and stalk (white bracket) of the indicated genotypes stained with antibodies to the follicle cell marker, FASIII (green), and with TO-PRO-3 iodide (red). A) *Df(3L)Cat*/+ (Control), B) *Capr-* showing a reduced primary stalk, and an aberrantly packaging egg chamber displaying an absence of stalk (dashed bracket) and misencapsulation of a single germline cell (arrow), and C) *Capr-* containing a disorganized stalk. D-E) Egg chambers stained for TO-PRO-3 (red). Optical sectioning revealed 16 nuclei in the control egg chamber (D), but fewer cells in a *Capr-* egg chamber (E) including nuclei of inappropriate size for this stage (arrowhead). Scale bar is 30 microns.

Examination of developing egg chambers revealed two additional defects in *Capr* mutant ovaries not observed in heterozygotes. First, a small portion of encapsulated egg chambers contained an inappropriate number of nuclei (Figure 2B, 2E, and Table S1). Occasionally, egg chambers were also observed that displayed heterogeneity in the size and presumed ploidy of the nuclei (Figure 2E) or portions of two cysts packaged into one egg chamber (data not shown). Similar defects have been reported for mutations in genes specifically affecting the FSC lineage [28], [29], [30], [32], [35], [38], but also resemble those attributed to GSC proliferation defects in *fmr1* mutants [39], [40]. In addition to the packaging defects, we observed occasional aberrations in cell number and/or organization of the stalk cells connecting developing egg chambers of *Capr-* ovarioles (Figure 2, compare brackets in A-C). Because stalk cells are exclusively derived from FSCs this suggests that at a minimum, *Capr* function is required by the FSC lineage, but could play a role in both the FSC and GSC lineages.

### *Capr* is Specifically Required for Maintenance of Follicle Stem Cells

**Figure 3:**
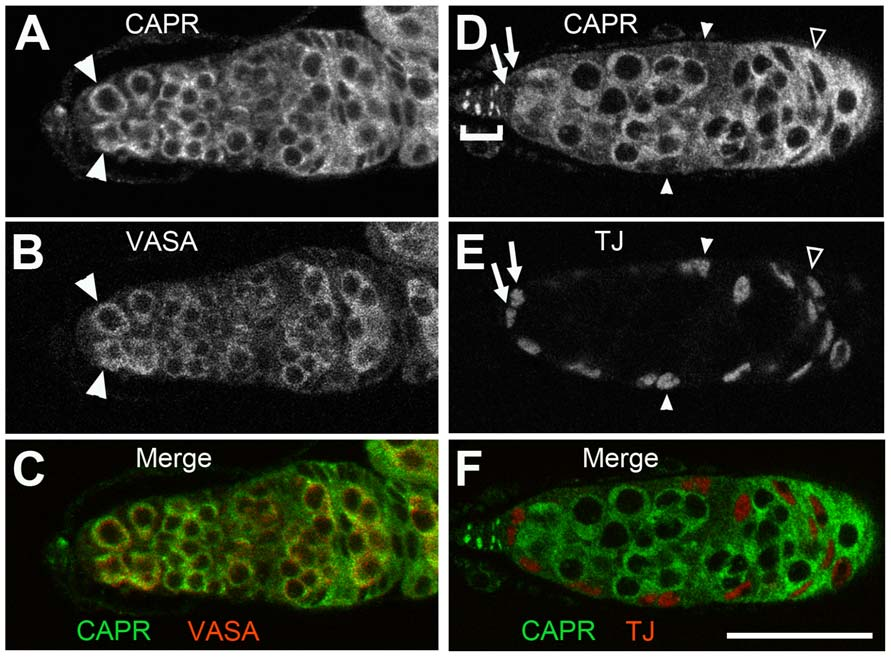
CAPR is present in both somatic and germline cells of the germarium. Immunofluorescence analysis of wild type germaria indicates CAPR is present in cells identified by the germline marker VASA (A-C) including the GSCs (arrowheads in A, B), and in some of the somatic cells identified by the nuclear protein Traffic Jam (TJ) (D-F). CAPR is present in the FSC-derived follicle cells (open arrowhead, D, E), and as bright cytoplasmic puncta in differentiated terminal filament cells (bracket in D), but is barely visible in the cap cells (arrows in D, E), and escort cells (closed arrowheads in D, E). Scale bar is 30 microns.

**Figure 4:**
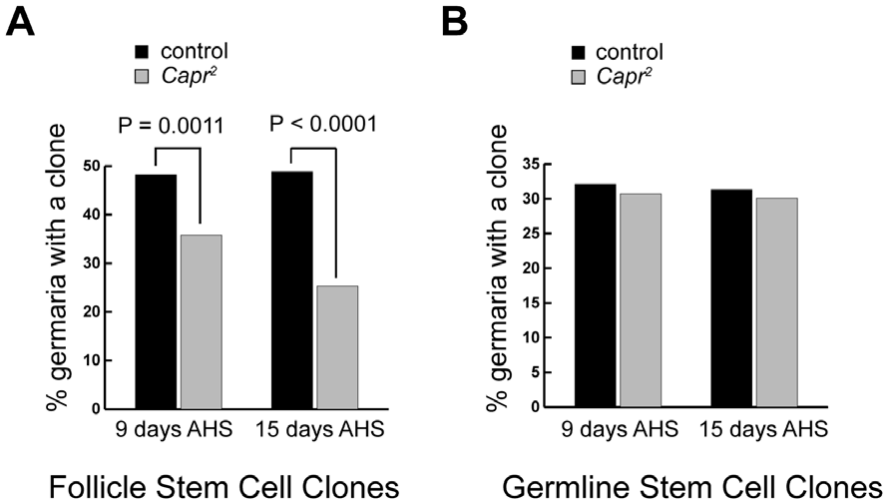
Loss of *Capr* leads to loss of follicle stem cells but not germline stem cells. The heat shock-FLP system was used to generate clones homozygous for the FRT80B (control) or for the FRT80B *Capr^2^* (*Capr^2^*) chromosome. Percent germaria containing follicle stem cell clones (A), or germline stem cell clones (B) were quantified at 9 and 15 days after heat shock (AHS). The number of germaria analysed for 9 day and 15 day data respectively was 106 and 166 for control and 78 and 166 for *Capr^2^*.

*Caprin* protein is found throughout all cells in the germarium. Using preabsorbed anti CAPR serum under conditions where staining is undetectable in *Capr* mutant ovaries (Figure S2) we observed relatively high CAPR expression in wild type ovaries in the GSC lineage, FSC lineage, and terminal filament compared to the cap cells and escort cells, where CAPR is barely detectable (Figure 3 and data not shown). To determine which lineage requires *Capr* function, we initially used the heat-shock-induced FLP/FRT method to generate marked homozygous *Capr* mutant clones in a heterozygous mutant female [41]. Mutant clones from either lineage showed no gross defects in size, morphology, or polarity as determined by immunostaining for Actin or the ß-catenin ortholog Armadillo (data not shown), suggesting that *Capr* is not required for anchoring FSC cells to their niche. During oogenesis, cells that are directly derived from an FSC division remain in the ovary for seven days. Following generation of *Capr* mutant clones, any *Capr* mutant cells detected in the ovary after seven days must be derived from a stem cell population that persisted after clone induction, while a reduction or absence of clones after seven days indicates a loss of the mitotically active stem cells [8]. The percentage of ovarioles containing either an FSC lineage clone or a GSC lineage clone was determined at various time points after clone induction. We found a statistically significant decrease in *Capr* mutant FSC-derived clones compared to the control (Figure 4A), and this difference increased with time after clone induction, indicating a progressive loss of mutant FSCs over time. In contrast, we observed no change in the frequency of *Capr* mutant GSC-derived clones compared to the control (Figure 4B). These results demonstrate that *Capr* is intrinsically required for FSC, but not GSC, maintenance. Since stalk cells and the follicle cells that encapsulate each cyst are derived from FSCs, all of the observed phenotypes are consistent with a role for *Capr* in both FSC maintenance and appropriate function of FSC progeny.

### *Capr* Specifically Regulates the Cell Cycle in the Follicle Stem Cell Lineage

The fates of stem cells and their progeny can be dramatically altered through changes in cell proliferation and cell cycle regulation [32], [42] and reviewed in [43]. FSC proliferation and differentiation are regulated by both *wg* and *hh* signals emanating from the cap cells at the tip of the germarium [28], [29], [32], [33]. We tested whether modulation of these signaling pathways could enhance or ameliorate the cyst packaging defects observed in *Capr* null germaria. A reduction in *wg* gene dosage, and consequent *wg* signaling, in a *Capr* mutant background caused a strong enhancement of the *Capr-* phenotype, such that all germaria contained supernumerary cysts in region 2a (Table 1). A similar reduction in *ptc,* which is expected to increase *hh* signaling and FSC proliferation [33], led to a reduction in supernumerary cysts in region 2a of *Capr-* germaria (Table 1). These results suggest that alterations in cell proliferation in the FSC lineage can specifically enhance or suppress the *Capr-* phenotype.

**Figure 5:**
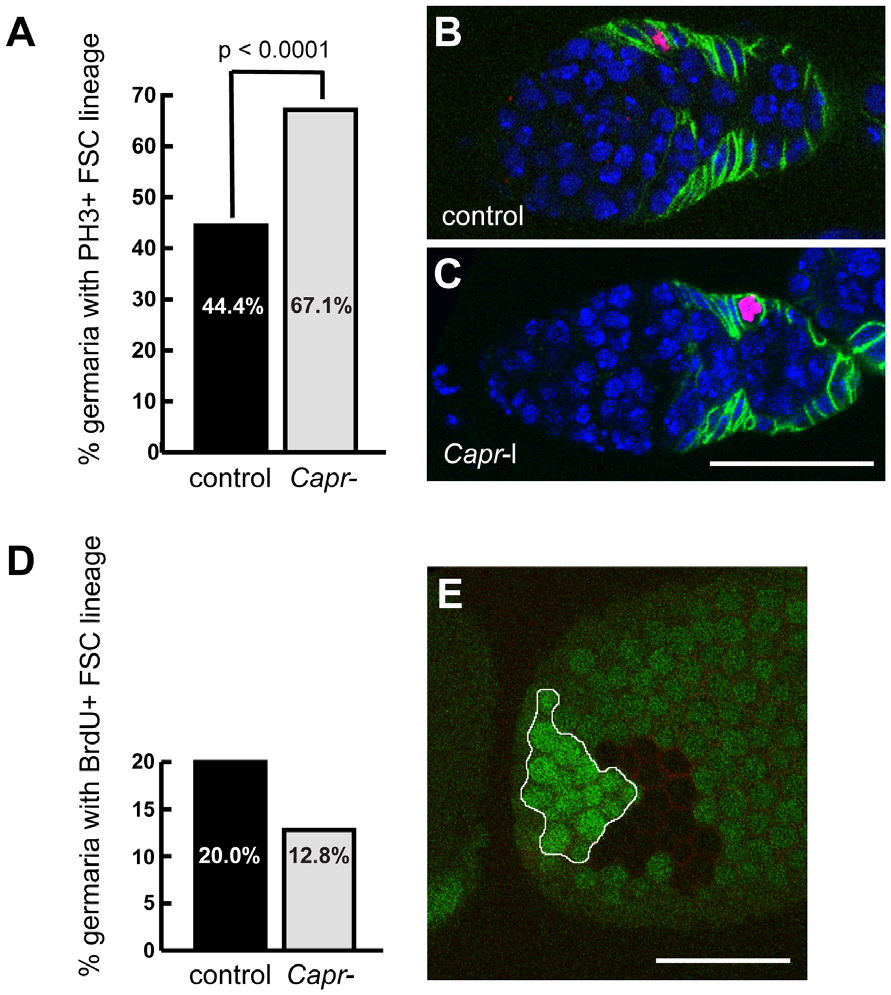
Loss of *Capr* alters cell cycle dynamics but not proliferation rates in the FSC lineage. A-C) Fixed *Df(3L)Cat/+* (control), or *Df(3L)Cat/Capr^2^* (*Capr-*) germaria were stained with antibodies to phospho-histone H3 (red), and FasIII (green), and with TO-PRO-3 iodide (blue). A) Quantification of the % of germaria scored that showed any phospho-histone H3-positive staining in cells of the FSC lineage. The number of germaria analysed was 81 control, 82 *Capr*-. B-C) Examples of stained germaria of the indicated genotypes. Size bar is 30 microns. D) Quantification of the % of pulse labeled germaria scored that incorporated BrdU in cells of the FSC lineage. The difference between *Df(3L)Cat/+* (control), or *Df(3L)Cat/Capr^2^* (*Capr-*) was not significant (P  =  0.09). The number of germaria analysed was 80 control, 86 *Capr*-. E) Tangential section of a fixed stage-10 egg chamber stained for GFP (green) and FasIII (red). The *Capr-* follicle cell clone (no GFP staining) and its adjacent wild-type twin-spot (bright green) are of similar size. Size bar is 60 microns.

Because *Capr* has been implicated in cell cycle regulation in both *Drosophila* and vertebrate cells [36], [44], [45], we considered the possibility that *Capr* might directly regulate the cell cycle in ovarian stem cells and their progeny. We used two approaches to determine whether loss of *Capr* alters the cell cycle in the FSC lineage: phospho-histone H3 staining and BrdU incorporation. Phospho-histone H3 specifically labels mitotic chromosomes [46], and is generally used to identify cells that are undergoing mitosis. We observed a statistically significant increase in the percentage of fixed *Capr*- germaria containing FSC-lineage cells in mitosis compared to the control germaria (Figure 5A). These results indicate that FSC-lineage cells in *Capr* mutant germaria are either undergoing more cell divisions or they are spending more time in mitosis. If the FSC-derived cells are undergoing more divisions there should be an equivalent increase in the number of cells in other phases of the cell cycle. We identified cells in S-phase by pulse labeling with BrdU, a thymidine analog incorporated into DNA during S-phase [47]. The percentage of *Capr-* germaria containing FSC-lineage cells in S-phase was not increased relative to controls (Figure 5D), and was in fact slightly reduced, although this difference was not statistically significant (p  =  0.09). This suggests that the defects found in *Capr* mutant ovaries are not due to alterations in overall proliferation rates, but are due to an alteration in lineage-specific cell cycle dynamics. Consistent with this interpretation, we did not find any striking differences in the overall size of the *Capr-* clones induced in heterozygotes compared to the simultaneously generated adjacent wild type clones (twin spots). This was true for clones observed in late stage egg chambers (Figure 5E) or recently induced within the germarium (data not shown). Together these data demonstrate that loss of *Capr* alters the cell cycle dynamics in the FSC lineage in a specific way, leading to prolonged mitosis but not an overall change in cycle length.

### *Capr* may Regulate CYCB Levels in the Follicle Stem Cell Lineage

CAPR is believed to act as a signal-dependent regulator of specific target mRNAs [36], [44], [48], [49]. In *Drosophila*, *Capr* modulates the translation of two mRNAs encoding cell cycle regulators during the mid-blastula transition: *CycB* and *frs* [36]. Of these known targets *frs* is not expressed in the germarium [50], [51], however, *CycB* is expressed in both the GSC and FSC lineage and is required for GSC divisions [52]. CYCB is a mitotic cyclin whose destruction is required to exit mitosis [53], making it a good candidate to mediate the alterations in the cell cycle we observe in *Capr*- ovarioles. Since CYCB levels oscillate during the cell cycle, we were unable to accurately compare CYCB levels directly by immunofluorescence. However, genetic manipulation of CYCB levels produced results consistent with a role for CYCB as an effector of the *Capr-* phenotype. Reducing the genetic dose of *CycB* in a *Capr* mutant background partially rescued the supernumerary cyst phenotype seen in *Capr-* germaria (Table 1) indicating that a critical level of CYCB is necessary to generate this phenotype. Furthermore, if the *Capr-* phenotype we observe is primarily due to an increase in CYCB, then overexpression of CYCB in a wild type ovary should also produce this phenotype. We tested this using *Act5C-GAL4* and *UAS-CycB* transgenes to drive *CycB* expression in all FSC lineage cells (data not shown). Overexpression of CYCB led to a specific increase in the number of cysts present in region 2a (Table 1) as was seen in *Capr*- germaria. Furthermore, a small percentage of ovarioles had stalk cell defects when CYCB was overexpressed (data not shown) suggesting that most if not all aspects of the *Capr* mutant phenotype can be explained by misregulation of *CycB*.

### *fmr1* and *Capr* Coordinately Regulate the Follicle Stem Cell Lineage

Previously, our lab showed that CAPR and dFMRP bind and regulate expression of some of the same mRNAs, including *CycB*, and that loss of *Capr* in a *fmr1* heterozygous background results in a more severe phenotype than loss of either gene alone ([36] and Figure S1). To date, *fmr1* has been implicated only in the maintenance and differentiation of GSCs, but not FSCs [37], [39], [40], [54]. However, the reported effects of *fmr1* on GSCs are not intrinsic, and dFMRP is expressed in somatic tissues, with the exception of the terminal filament cells which lack detectable dFMRP (data not shown, consistent with [37], [39]). We asked whether *fmr1* might also play a role in the FSC-dependent packaging of cysts. Complete loss of *fmr1* alone produced a minimal increase in unencapsulated cysts (18.6% of germaria showing >5 cysts in region 2a) compared to the 85.6% seen in *Capr* null germaria (Table 1). In a *Capr* mutant, however, even partial reduction of *fmr1* generated unencapsulated cysts in 100% of the germaria (Table 1). Because loss of *fmr1* has no effect on cyst production by GSCs [37] the genetic interaction between *Capr* and *fmr1* suggests that *Capr* and *fmr1* coordinately regulate cyst encapsulation by the FSC lineage.

## Discussion

Stem cells are influenced by a combination of intrinsic and extrinsic factors instructing them to produce more stem cells and/or differentiating progeny (reviewed in [1], [11], [55]). Translational regulation has proven to be of fundamental importance in control of GSC identity and behavior, but surprisingly little is known about the relative importance of this mode of regulation in controlling the fate of FSCs. Here we report that the translational regulator *Capr* functions as an intrinsic factor required for the proper maintenance of FSCs, and that loss of *Capr* disrupts cell cycle dynamics within the FSC lineage. We propose that *Capr* is required for proper execution of the cell cycle in the FSC-lineage, in part through modulation of CYCB protein levels. In this model misregulation of the *Capr*-dependent cell cycle leads to defects in somatic cell differentiation, with a concomitant disruption of the ability to correctly package developing cysts into egg chambers. The ability of *fmr1* mutation to enhance the encapsulation defects implicates these two translational regulatory factors in coordinate control of this aspect of ovary function.

### Is *Capr* solely Required in the FSC Lineage?

Given the similarities between the *Capr* mutant phenotype and the mutant phenotype of genes involved in FSC proliferation, maintenance, and differentiation, it is possible that *Capr* is only required in the FSC lineage. Our data, however, cannot rule out the possibility that *Capr* is additionally required in non-FSC lineage cells to send extrinsic signals that impact the encapsulation process. For example, our clonal analysis demonstrated that *Capr* is not required for GSC maintenance. However this technique cannot rule out a requirement for *Capr* in the GSC lineage for other functions such as cell-cell communication. Similarly, because *Capr* protein was barely detectable in the cap cells or the escort cells it seems less likely that *Capr* has a critical function in these populations, but not impossible. A more appealing candidate population might be the terminal filament cells based on their prominent CAPR-containing puncta. Terminal filament cells are known to function along with the cap cells as niche cells for both the GSCs and FSCs (reviewed in [1]). It will be interesting to determine whether the bright puncta of CAPR we observe in the terminal filament cells represent ribonucleoprotein structures involved in signal-responsive translational regulation similar to the CAPR-containing neuronal and stress granules of vertebrates [44], [49], [56]. Ultimately, because the clonal removal of *Capr* specifically from the FSC’s alone disrupted stem cell maintenance, the simplest interpretation of the current data is that an intrinsic role for *Capr* in the FSC’s can account for all the phenotypes observed. Further study will be required to determine whether *Capr* has additional roles in other ovarian cells.

### *fmr1* Collaborates with *Capr* in the Ovary

During *Drosophila* embryogenesis, *Capr* is known to functionally collaborate with *fmr1* to regulate the timing of the mid-blastula transition [36]. The functional interaction of these two translational regulators is further supported by evidence that CAPR and dFMRP coimmunoprecipitate from *Drosophila* embryos [36] and associate with common ribonucleoprotein structures such as neuronal granules [48], [56], [57], [58], stress granules [44], [59], [60], [61], *Drosophila* lipid droplets [62], and the 5' cap structure of mRNAs in the ovary [63]. In the ovary *Capr* and *fmr1* are expressed in both the germline and somatic cells (this work and [39]). A role for *fmr1* in somatic cells and encapsulation was initially considered unlikely because *fmr1* mutant egg chambers displaying germ cell proliferation defects are surrounded by apparently normal follicle cells, and are typically flanked by appropriately packaged egg chambers [39], [40]. The maintenance of GSCs, however, relies on *fmr1* function outside the GSCs [37], [40] leaving open the possibility that *fmr1* functions in the germline cysts and somatic cells of the ovary. Our data indicate that *fmr1* and *Capr* genetically interact to regulate cyst encapsulation and female fecundity. One possible interpretation of our data is that CAPR and dFMRP co-regulate translation of a set of transcripts in FSCs or their progeny important for cyst encapsulation. Alternatively, CAPR and dFMRP could individually regulate distinct transcripts required for proper FSC function. In either case both translational regulators are necessary for proper encapsulation of developing cysts and generation of a functional egg chamber.

Intriguingly recent studies have indicated that FMRP is required for normal functioning of the human ovary as well. Although the mechanism has yet to be determined, FMR1 premutation carriers with no neuro/psychiatric symptoms nevertheless show reduced fecundity due to aberrant control of follicular recruitment and ovarian reserves [64]. In addition to its role in the ovary, dFMRP is reported to affect proliferation of Sertoli cells, the niche cells of the male gonad [65], [66], and to regulate stem cell behavior in the nervous system (reviewed in [67]) and it will be interesting to determine whether CAPR also participates in these processes.

### *Capr* may Act as a Cell Cycle-specific Translational Regulator in the Ovary

In stem cells control of the cell cycle may be uniquely linked to cell fate. For example, in mouse neuroepithelial cells, simply altering the length of G_1_ using cyclin-dependent kinase inhibitors induces differentiation [68]. Likewise in the ovary, as cells produced by FSCs proceed through successive divisions they acquire longer S-phases and increased epigenetic stability, conditions which promote the differentiated state [69]. Furthermore, elevated levels of CYCE are required in the FSC’s themselves, to promote the adherence of these stem cells to their niche [42]. It is therefore plausible that even subtle modulation of the cell cycle by *Capr* could have profound consequences for production of a functional egg chamber.

Translational control of cell cycle regulation is a specific mechanism reported to affect behavior of both GSCs and FSCs [21], [22]. Although CAPR is reported to be a signal-dependent regulator of translation in the vertebrate nervous system [48], [49], [56] it has been equally implicated in developmental regulation of proliferation: Caprin-1 levels correlate with cell proliferation states in many vertebrate tissues, and *caprin-1* deficient cells show a specific delay in G_1_-S progression [45], [70]. Similarly, FMRP has been predominantly studied because of its role in the nervous system where loss of FMRP causes mental retardation and autism (reviewed in [71]). However, loss of FMRP also generates significant aberrations in proliferation in both the ovary and testis [40], [66]. The encapsulation defects we see, therefore, could be due entirely to a *Capr*- or *Capr* and *fmr1*-dependent alteration of the cell cycle in the FSC lineage.

CAPR is a sequence-specific RNA-binding protein believed to function by altering translation and/or localization of specific mRNA targets [36], [44], [48], [49], [56]. However, despite our genetic evidence that *CycB* misregulation underlies the defects we observed, CAPR may regulate other mRNAs, and the phenotype we see could be due to a cumulative misexpression of mRNAs involved in cell cycle control and other processes. In this regard there is still much to learn about how CAPR or FMRP achieve temporal and target specificity. For example, both *Capr* and *CycB* are expressed in GSCs and numerous other tissues but *Capr* does not appear to regulate *CycB* in all of these. Future determination of all relevant mRNA targets in the ovary, and the mechanism for regulating CAPR function and specificity would be constructive steps towards understanding the role of translational regulation in the control of stem cell behavior.

## Materials and Methods

### Fly Stocks

Stocks were reared on standard cornmeal media. *Df(3L)Cat ri fmr1^3^* and *Capr^2^* were previously described [36]. The *FRT80B Capr^2^* stock was generated for this paper. *Df(3L)Cat ri sbd^1^ e, Df(3R)Exel6265, P[+mC]  =  XP-U}Exel6265, FRT80B*, *hsFLP; FRT80B arm-lacZ*, *FRT80B ubi-GFP*, *wg^l–12^*, *ptc^S2^*, *CycB^2^*, *UAS-CycB*, and *Act5C-GAL4* stocks were obtained from the Bloomington Stock Center (Bloomington, IN).

### Immunofluorescence

Ovaries were from females fed fresh yeast paste for a minimum of two days. Ovaries were dissected into PBS on ice and broken up by pipeting. Samples were fixed 20 minutes in 4% formaldehyde in PBS, followed by four 15 minute washes in PBST (PBS + 0.1% Triton-X 100). Fixed samples were blocked with PBTA (PBST + 1% BSA) for 1–2 hours at room temperature, incubated with primary antibody in PBTA overnight at 4°C, washed with PBST as above, and incubated 2 hours with secondary antibody. Samples were washed with PBST as above and mounted in Vectashield (Vector Laboratories). Anti-Caprin polyclonal serum was preabsorbed against Oregon-R ovaries in PBTA prior to use.

Primary Antibodies: mouse anti-FasIII (1∶50, 7G10) and mouse anti-Slit (1∶25 C555.6D) were from the Developmental Studies Hybridoma Bank, guinea pig anti-Traffic Jam (1∶3000, [72]), rabbit anti-phospho-Histone H3 (Ser10) (1∶500, Millipore 06–570), mouse anti-BrdU (1∶20, Becton Dickinson 347580), and rabbit anti-Caprin (1∶500, [36]).

Secondary Antibodies: Alexa Fluor 488 goat anti-rabbit IgG (1∶500), Alexa Fluor 488 goat anti-mouse IgG (1∶500), Alexa Fluor 546 goat anti-rat IgG (1∶500), Alexa Fluor 633 goat anti-rabbit IgG (1∶500), Alexa Fluor 633 goat anti-mouse IgG (1∶500), and Alexa Fluor 546 goat anti-rabbit IgG (1∶500) were from Invitrogen. DNA was visualized with TO-PRO-3 iodide (1∶2000, Invitrogen).

### Clonal Analysis

*hsFLP; FRT80B ubi-GFP/FRT80B Capr^2^* or *hsFLP; FRT80B arm-lacA/FRT80B Capr^2^* flies were transferred to well yeasted vials each day for at least two days before heat-shock treatment. Flies were then heat-shocked in a 38°C running water bath for 1 hour (twin-spot analysis) or for 1 hour on three consecutive days (FSC and GSC clonal analysis). Flies were then transferred to well-yeasted vials every day until ovaries were dissected and prepared for immunofluorescence.

### BrdU labeling

Flies were labeled essentially as described [47]. Briefly, ovaries from well-fed flies were dissected into room temperature Schneider’s Insect Medium (Sigma S0146). Medium was replaced with Schneider’s Insect Medium containing 10 mM 5-Bromo-2-Deoxy-Uridine (Roche 10280879001), and the ovaries were incubated for 1 hour on a nutator at room temperature. Ovaries were washed in Schneider’s Insect Medium twice for three minutes each, and fixed 20 minutes in 1∶1∶4 37% formaldehyde: Buffer B (100 mM KH_2_PO_4_/K_2_HPO_4_ pH 6.8, 450 mM KCl, 150 mM NaCl, 20 mM MgCl_2_): H_2_O. Fixed samples were washed twice in PBST and twice in DNase buffer (66 mM Tris-HCl pH 7.5, 5 mM MgCl_2_, 1 mM 2-mercaptoethanol) for 15 minutes each, incubated in DNase buffer with 12.5 U/ml DNaseI (Fermentas EN0521) at 37°C for 30 minutes, and washed three times with PBST for 10 minutes each. Samples were blocked in PBTA for 30–60 minutes, incubated in PBTA containing anti-BrdU antibody overnight at 4°C, and washed four times for 15 minutes each in PBTA. Secondary antibody incubation and subsequent steps were as for immunofluorescence.

#### Statistics

X^2^ analysis (http://graphpad.com/quickcalcs/chisquared2.cfm) was performed using 1 degree of freedom where a two-tailed P value of < .05 was deemed significant. For clonal analyses at 9 days values for GSCs were X^2^ = 0.06, P  =  0.81 and for FSCs X^2^ = 10.70, P  =  0.001. For clonal analyses at 15 days values for GSCs were X^2^ = 0.10, P  =  0.75 and for FSCs X^2^ = 36.67, P < 0.0001. For phopho-Histone H3 staining values were X^2^ = 17.09, P < 0.0001. For BrdU incorporation values were X^2^ = 2.79, P  =  0.09.

## Supporting Information

Figure S1 ***Capr*** **null flies with reduced** ***fmr1*** **show reduced fecundity over time.** Well fed females of the indicated genotypes were mated to Oregon R males and eggs were collected from females of the indicated age range. *Df* refers to the *Capr* deficiency, *Df(3L)Cat*. n  =  total eggs collected. A) Graph showing eggs laid per unit time per female. B) Graph of the percent of eggs that hatched. Error bars depict standard deviation. Note the dramatic decrease in egg production and viability in 9–11 day old *Df, fmr1^3^/Capr^2^* females.
(TIF)

Figure S2 **Polyclonal anti-CAPR antibodies used in this study show no background staining in the germarium.** Representative germaria from A) Oregon R (control) or B) *Capr2/Df(3L)Cat* (*Capr-*) flies stained with preabsorbed anti-Caprin antibodies (top panels, CAPR, green) and TO-PRO-3 iodide (bottom panels, DNA, red). Scale bar is 30 microns.
(TIF)

Table S1 **Data are shown for the percent of ovarioles containing an egg chamber with the indicated number of nurse cells.** *Df* refers to the *Capr* deficiency, *Df(3L)Cat*. n  =  number of ovarioles scored.
(DOCX)
