# Supplementary figures and images for: Caprin Controls Follicle Stem Cell Fate in the Drosophila Ovary
Source: PLoS One. 2012 Apr 6;7(4):e35365. doi: 10.1371/journal.pone.0035365 (PMC3320888; doi:10.1371/journal.pone.0035365)

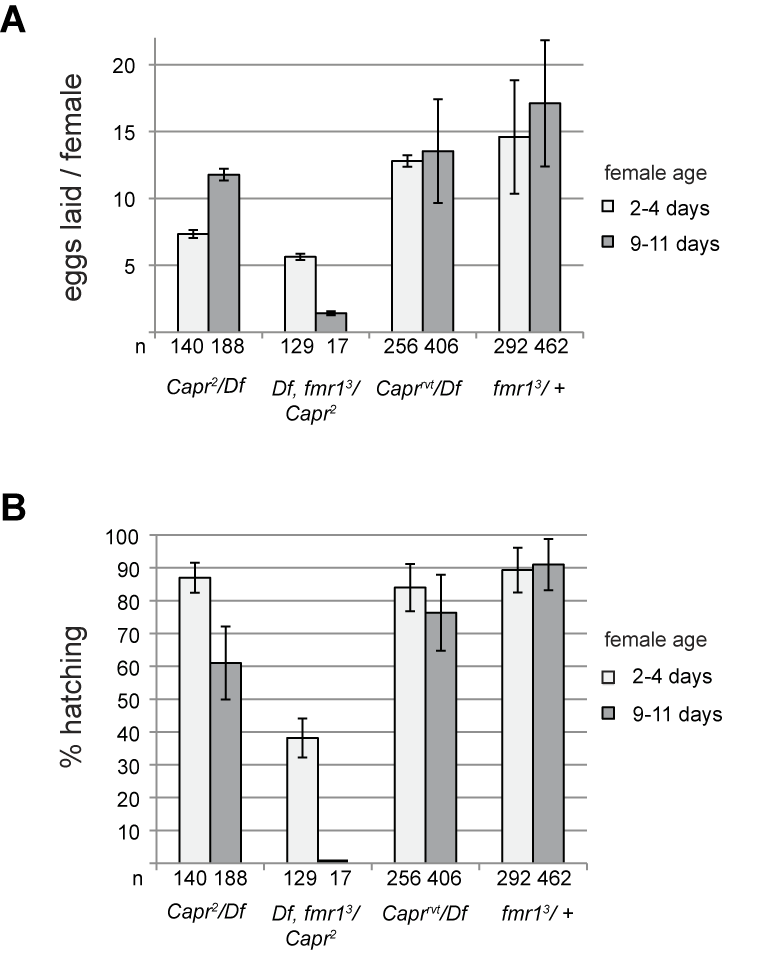

Supplement: Figure S1 — Capr null flies with reduced fmr1 show reduced fecundity over time. Well fed females of the indicated genotypes were mated to Oregon R males and eggs were collected from females of the indicated age range. Df refers to the Capr deficiency, Df(3L)Cat. n = total eggs collected. A) Graph showing eggs laid per unit time per female. B) Graph of the percent of eggs that hatched. Error bars depict standard deviation. Note the dramatic decrease in egg production and viability in 9–11 day old Df, fmr13/Capr2 females. (TIF) [file pone.0035365.s001.tif]

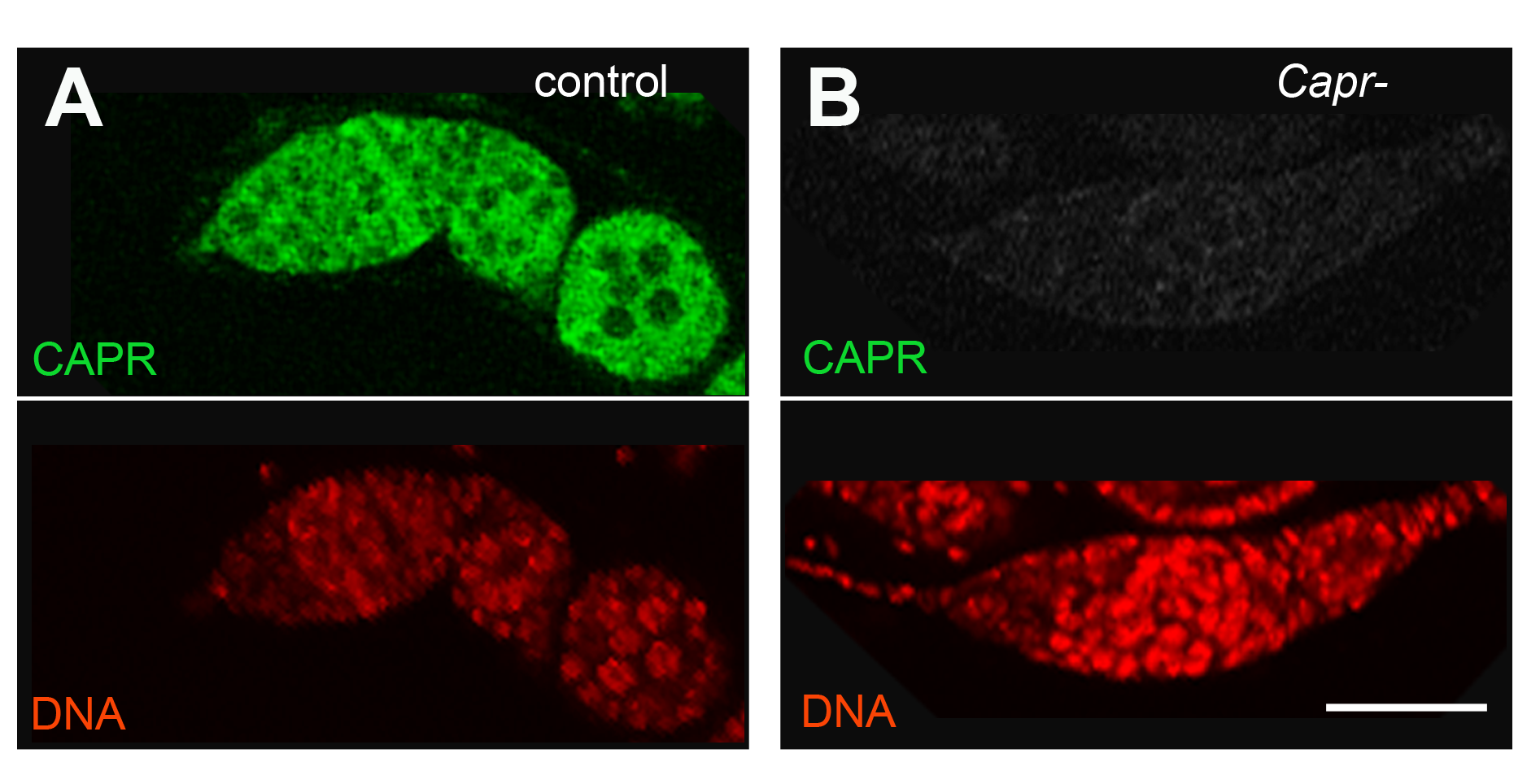

Supplement: Figure S2 — Polyclonal anti-CAPR antibodies used in this study show no background staining in the germarium. Representative germaria from A) Oregon R (control) or B) Capr2/Df(3L)Cat (Capr-) flies stained with preabsorbed anti-Caprin antibodies (top panels, CAPR, green) and TO-PRO-3 iodide (bottom panels, DNA, red). Scale bar is 30 microns. (TIF) [file pone.0035365.s002.tif]
