# Supplementary material for: Caprin Controls Follicle Stem Cell Fate in the Drosophila Ovary
Source: PLoS One. 2012 Apr 6;7(4):e35365. doi: 10.1371/journal.pone.0035365 (PMC3320888; doi:10.1371/journal.pone.0035365)
Supplement: Table S1 — Data are shown for the percent of ovarioles containing an egg chamber with the indicated number of nurse cells. Df refers to the Capr deficiency, Df(3L)Cat. n = number of ovarioles scored. (DOCX) [file pone.0035365.s003.docx]

Table S1

Loss of *Capr* produces egg chambers with aberrant numbers of nurse cells.

| Genotype | >15 nurse cells | 15 nurse cells | n |
| --- | --- | --- | --- |
| *Capr^2^/Df* | 4.3 % | 95.7 % | 70 |
| *Df, fmr1^3^/Capr^2^* | 24.4 % | 75.6 % | 41 |
